# Oral Cooling and Carbonation Increase the Perception of Drinking and Thirst Quenching in Thirsty Adults

**DOI:** 10.1371/journal.pone.0162261

**Published:** 2016-09-29

**Authors:** Catherine Peyrot des Gachons, Julie Avrillier, Michael Gleason, Laure Algarra, Siyu Zhang, Emi Mura, Hajime Nagai, Paul A. S. Breslin

**Affiliations:** 1 Monell Chemical Senses Center, Philadelphia, PA, United States of America; 2 AgroSup Dijon Institut National Superieur, Dijon, France; 3 AgroParisTech Paris, Paris, France; 4 Suntory Global Innovation Center Limited, Osaka, Japan; 5 Rutgers University Department of Nutritional Sciences, New Brunswick, NJ, United States of America; Barnard College, UNITED STATES

## Abstract

Fluid ingestion is necessary for life, and thirst sensations are a prime motivator to drink. There is evidence of the influence of oropharyngeal stimulation on thirst and water intake in both animals and humans, but how those oral sensory cues impact thirst and ultimately the amount of liquid ingested is not well understood. We investigated which sensory trait(s) of a beverage influence the thirst quenching efficacy of ingested liquids and the perceived amount ingested. We deprived healthy individuals of liquid and food overnight (> 12 hours) to make them thirsty. After asking them to drink a fixed volume (400 mL) of an experimental beverage presenting one or two specific sensory traits, we determined the volume ingested of additional plain, ‘still’, room temperature water to assess their residual thirst and, by extension, the thirst-quenching properties of the experimental beverage. In a second study, participants were asked to drink the experimental beverages from an opaque container through a straw and estimate the volume ingested. We found that among several oro-sensory traits, the perceptions of coldness, induced either by cold water (thermally) or by l-menthol (chemically), and the feeling of oral carbonation, strongly enhance the thirst quenching properties of a beverage in water-deprived humans (additional water intake after the 400 ml experimental beverage was reduced by up to 50%). When blinded to the volume of liquid consumed, individual’s estimation of ingested volume is increased (~22%) by perceived oral cold and carbonation, raising the idea that cold and perhaps CO_2_ induced-irritation sensations are included in how we normally encode water in the mouth and how we estimate the quantity of volume swallowed. These findings have implications for addressing inadequate hydration state in populations such as the elderly.

## Introduction

The sensation of thirst is a warning, a signal from interoceptors indicating the need to re-hydrate. It plays a key role in the maintenance of body fluid homeostasis by motivating animals to seek and ingest water (as well as to ingest salt to retain the water). It is generally believed that drinking fluids diminishes thirst because it leads to rehydration and consequently reduced physiological thirst signals. But thirst is quenched long before ingested liquids are absorbed and equilibrated with body fluids. The oral sensations of drinking appear to participate in thirst quenching and the termination of water intake pre-absorptively [1]. Cognitive projections of body hydration during drinking are important both to circumvent overhydration and ensure that physiological needs will be met. Yet, how ingested liquids are metered orally and what sensory cues are involved is largely unexplained.

Thirst can be stimulated physiologically by: small increases (1–2%) in plasma osmolality (pOsm), marked decreases in plasma volume (5–8% loss in body fluid volume), and manipulation of fluid regulatory hormones. During water deprivation and subsequent dehydration, both increased pOsm and decreased plasma volume may occur. The restoration of total body water is controlled by a complex feedback system of inhibitory and excitatory osmoregulatory signals and hormones.

A principal water regulatory system is the renin-angiotensin-aldosterone system (RAAS), which acts on blood pressure and fluid balance both directly and indirectly via hormones, including aldosterone and arginine vasopressin (AVP). The released aldosterone increases the reabsorption of sodium in the kidneys, which in turn results in the retention of water. And the released AVP acts on the kidneys to enhance the reabsorption of water at the nephron in order to dilute the increased osmolality and retain water [2]. If the hormonally-induced reabsorption of water is not sufficient, then the sensation of thirst is stimulated to prime water seeking and drinking behavior and adequately decrease pOsm and increase plasma volume [3]. In humans, above a certain osmotic threshold there is a linear relationship between the increases in pOsm, AVP concentration, and reported thirst [4]. The relationships among these three regulators of hydration have been exploited to demonstrate the existence of pre-absorptive mechanisms in the regulation of body fluid homeostasis [5,6]. For example, in humans a steep and sudden decrease of AVP concentration after drinking, concomitant with a reduction of thirst [7,8], is observed within minutes, well before ingested water has emptied from the stomach or pOsm returned to baseline.

Although those anticipatory responses are likely dependent on a combination of afferent inputs from oropharyngeal and gastric receptors, the rapid inhibition of thirst and AVP secretion appear to be principally a response to oropharyngeal sensory stimulation [9,10] because they are not observed when the oropharyngeal receptors are bypassed. Moreover, the oral and pharyngeal regions appears to contribute to the accurate replacement of body fluids by metering the volume of ingested fluid, as demonstrated in dehydrated dogs, and subsequently in humans, which drink in proportion to water deficits, even if the ingested water is removed from their stomach [11,12]. Human studies have further shown that the rapid fall in plasma AVP observed after drinking (10 to 15 ml/kg) cannot be provoked when merely gargling [5], nor by the ingestion of very small quantities of water (1 ml/kg); rather full stimulation from drinking is required [13]. However, sucking on ice chips for 30 min, in contrast to consuming an equivalent small volume of water (100 mL) at 25°C, causes a decrease in thirst and a prompt and sustained fall in plasma AVP [14]. Therefore, activation of cold signals from the oropharynx appears to play a role in pre-absorptive water intake signaling. This finding is supported by the observation that dehydrated people have an increased desire for cold liquids. But the basis for the preference for cold beverages remains unclear. At equal volumes both room temperature and cold water would be expected to quench thirst equally. One explanation for why they do not could be that oral cooling is a signal of drinking [15], since water typically appears cool when ingested because it is usually several degrees below the temperature of the oral cavity.

In our study, we examined which sensory trait(s) and oral qualities influence levels of thirst, and how they impact the thirst quenching efficacy of ingested liquids. We focused on demonstrating which oral-sensory cues influence projections of body hydration, influence volumes consumed, and affect the perception of the amount consumed.

## Materials and Methods

### Study on respective efficacies of sensory trait(s) manipulated in experimental beverages on thirst reduction

#### Participants

A total of 98 subjects (58 females and 40 males) between 20 and 50 (mean 28.1 29.4) years of age were recruited from the Philadelphia area and were paid to participate. All were healthy with no known taste or smell deficiency. Pregnant women and people under medication (diuretics) or with diseases (influenza, oral surgery, dialysis…) that are known to interfere with taste and fluid intake were excluded from the study. The study protocol was approved by the Internal Review Board of the University of Pennsylvania and subjects gave written informed consent prior to participation.

#### Stimuli

Beverages at different temperatures: Distilled water beverages with a pH of 5.4 were stored in glass containers prior to the study at two different temperatures: 20–22°C and 6°C. The 6°C temperature was chosen because it is the approximate temperature of a drink served from a refrigerator. The 21°C temperature was chosen because it corresponds to the temperature of a drink that is served at room temperature (RT), but it is noticeably cooler than the inside of the mouth (~33°C). Beverage temperatures were confirmed with a digital thermometer throughout the study. Acidified beverage: 5 mM (0.96g/l) citric acid was added to distilled water. The acidified solution had a pH of 4.0 and was kept cold (6°C). Carbonated beverage: The carbonated water used was a commercially available soda water (Vintage Seltzer water®). This water had a pH of 4.2; 8 g/L of CO_2_ and was kept cold at 6°C or 20–22°C. One bottle was used per session and was opened just before drinking to maintain CO_2_ level consistency between sessions. Astringent and sweet beverages: For the astringent beverage, 0.75 g/L of GSE (Grape Seed Extract, Trader Joe’s) was prepared from a stock solution of 3 g/L of GSE, renewed every 3 days. Astringency was manipulated because most humans ingest tea with meals. The 0.75 g/L GSE solution was presented alone or mixed with 150 mM sucrose. Sweetness was manipulated because humans often ingest sweetened beverages. 150 mM sucrose diluted in distilled water was also tested without GSE. Pre-treatment solutions: For the menthol pre-treatment, a solution containing 0.04% dl-menthol (Sigma-Aldrich) was prepared in distilled water containing 2% (v/v) ethanol and 0.5% (w/v) polysorbate 80 (Tween 80, Sigma-Aldrich). Menthol crystals were first dissolved in ethanol, mixed with polysorbate 80 and brought to volume with dH_2_O. The control pre-treatment was an aqueous solution containing 2% ethanol and 0.5% polysorbate 80 (Tween 80, Sigma-Aldrich). The pre-treatment solutions were presented at room temperature. The menthol was employed to elicit the illusion of cold chemically.

#### Experimental protocol

Subjects were asked to refrain from eating and drinking for twelve hours before the test session. Upon arrival at the laboratory, they were weighed and asked to eat a standardized breakfast consisting of white bread toast (1 slice under 75 Kg body weight then an extra ½ slice per additional 25 Kg), spread with jelly, which all subjects consumed; no liquid was offered at this time. The purpose of this standardized breakfast was to increase thirst further. After a 30 minute period of rest in the testing room, subjects were asked to evaluate the level of their thirst on a computerized labeled magnitude scale (LMS). Then they drank 400 mL of an experimental beverage. They were asked to drink the whole volume in less than 5 min. After a 5 min rest period, participants were offered an excess of RT, still, unflavored water to drink from a jug until they no longer wished to continue. The volume of RT water consumed from the jug was measured and recorded.

For the experiment using the cooling effect of menthol, the same protocol as above was used with the following modifications: (1) the 400 mL samples were preceded by a pre-treatment of five cups of 5 mL solutions with or without 0.04% l-menthol, swished for 20 seconds and swallowed, repeated every minute, five times (for a total of 5 min of 25 mL of pretreatment), (2) the RT water jug was offered 15 min after the 400 mL experimental beverage, instead of 5 min after, in order to eliminate the menthol cooling effect on the RT water from the test jug, and (3) we asked participants to rate the perceived coldness of the experimental beverage to validate the effects of the menthol pre-treatment.

All the experimental beverages were tested in duplicate to assess reliability. Each experiment was performed by a different group of subjects.

### Study on estimated volume of experimental beverages ingested when blind to volume

#### Participants

10 individuals (9 females and 1 male) between 22 and 48 (mean 31.2) years of age were recruited from the Monell Chemical Senses Center and were paid to participate. All were healthy with no known taste or smell deficiency. Pregnant women and people under medication (diuretics) or with diseases (flu, oral surgery, dialysis…) that are known to interfere with taste and fluid intake, were excluded from the study. The study protocol was approved by the Internal Review Board of the University of Pennsylvania and subjects gave written informed consent prior to participation.

#### Stimuli

Three beverages were tested in the first study (RT water, cold water, and cold-carbonated water as described in the first experiment). RT and cold water were used in the second study.

#### Experimental protocol

Participants were asked to estimate the ingested volume of different beverages while they were unable to see the liquid or feel the beverages weight. All beverages were presented in lidded, opaque cups, at three different volumes (157.8, 251.5 and 400.9 mL). For each separate test, the whole sample was ingested through an opaque straw without holding the cup and the time of completion was recorded. For the first experiment, participants could drink the beverages at their own pace. For the second experiment, participants did the same task with RT water but at an experimenter-determined rate (slow or fast). The drinking rate was established by asking individuals to sip once and swallow at each tone of a digital metronome. The fast rate was set at 20 beats per minute (bpm) and the slow rate at 10 bpm. To judge the perceived volumes ingested, participants were presented with a series of 25 clear plastic cups with increasing volumes inside (from 125 to 506.1 mL in 6% volumetric increments) and were asked to choose the cup that most accurately represented the volume they just drank. Each condition was tested in triplicate.

#### Statistical analyses

Arithmetic means of intensity ratings or estimated volumes were calculated across the replicates within subjects and were used for statistical analyses. Statistical significance was evaluated using Student’s paired *t*-tests for each experiment, since they were run as independent experiments with different groups of subjects at different times. Statistical significance was set at *P* value <0.05. Fisher LSD ANOVA model was used in the study on estimated volume of beverage ingested, using IBM SPSS statistics 23, with a criterion of p < 0.05.

## Results

### Study on respective efficacies of sensory trait(s) in experimental beverages on thirst reduction

#### Thirst quenching effects of a physically cold beverage

**Fig 1:**
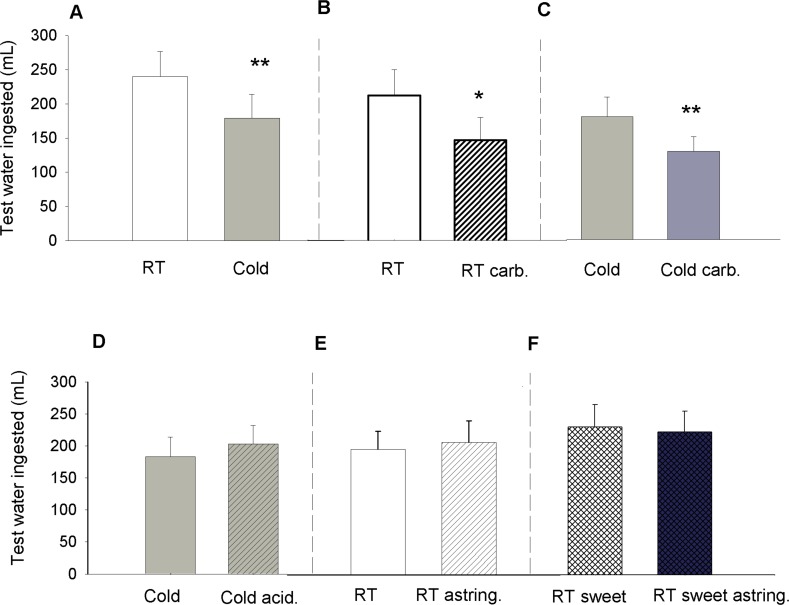
Effects of oral sensory stimulations on thirst quenching. Figures display the volume of extra room temperature (RT) water consumed after a 400 mL experimental beverage. Legends correspond to the experimental beverage tested. Each inset on the figure displays the results of different experiments and each experiment was performed by a different group of participants. (A) (n = 20 participants): RT water (20–22°C) vs Cold water (6°C). (B) (n = 20): RT water vs RT carbonated water (C) (n = 15): Cold water vs Cold carbonated water. (D) (n = 15): Cold water vs Cold acidified water. (E) (n = 15): RT water vs Astringent water. (F) (n = 18): RT Sweet water vs RT Sweet Astringent water. Data are represented as mean +/- SEM. ** indicates statistical significance at p< 0.01 and * p<0.05.

We first investigated the effects of cold water (6°C) vs room temperature (RT) water (20–22°C) on the thirst reduction. Participants deprived of food and water overnight first evaluated their thirst level on a labeled magnitude scale (LMS). Then they drank 400 mL of an experimental beverage (either cold water or RT water). After a 5 min rest period, participants were offered an excess of RT water to drink from a jug until they no longer wished to continue, which was within 15 minutes. The volume of RT water consumed from the jug was measured and recorded. Self-reported thirst ratings were used to verify that on average participants started the sessions with comparable levels of thirst (mean value was 35, just above the descriptor “strong” on an LMS). No differences in initial thirst levels were observed between sessions and experiments. Overall, participants drank significantly less RT water from the jug after a previous cold water beverage than after a RT water beverage (Fig 1A, p = 0.001). Thus, we infer that the cold water reduced thirst more effectively than did the RT water. All raw data may be found in Supplemental Information as Copy of Thirst Study Raw Data.

#### Thirst quenching effects of a carbonated beverage

A RT still water beverage was compared to a RT, carbonated water beverage. Overall, participants drank significantly less water from the jug after the RT carbonated water beverage than after the RT still water (Fig 1B, p = 0.0108). Thus carbonation at RT also reduces thirst. To examine if carbonation sensation and cold might have an additional effect on thirst, we then compared a cold, still water beverage to a cold, carbonated water beverage. Participants drank less from the jug after the cold carbonated water. Thus, carbonation further reduces thirst beyond the effects of cold water (Fig 1C, p = 0.004).

#### Thirst quenching effects of a physically cold and mildly acidified beverage

A slightly sour and cold water beverage with pH matched to the carbonated water (5 mM citric acid, pH = 4.0) was compared to a plain, cold water beverage. In these conditions, participants drank a similar quantity of RT water from the test jug after either experimental beverage (Fig 1D, p = 0.43). Thus, mild acidification does not reduce thirst beyond the thirst quenching effects of cold water and pH at this level likely does not explain the thirst quenching effects of carbonation.

#### Thirst quenching effects of astringent and sweet beverages

We next tested two additional oral sensory traits commonly found in beverages: astringency and sweetness. Astringency was elicited by 0.75 g/L grape seed extract (GSE) and sweetness by 280 mM sucrose. Both attributes were tested at RT and at levels of intensity perceived as strong when alone and as moderate when in mixture (based on LMS intensity ratings). In all tests, participants drank a similar quantity of RT water from the test jug after consuming the experimental beverages (Fig 1E and 1F). No statistically significant differences were found (p = 0.7 and 0.6 respectively). Thus, astringency and sweetness sensations neither reduced nor increased thirst beyond the thirst quenching effects of a RT beverage. These results reinforce the specificity of the effects of cold and carbonation on thirst reduction. The negative results found with sweet and astringent experimental beverages further indicate that response biases resulting from palatability effects, hedonic contrast, or demand characteristics of the study are not driving our results. We confirmed this in a supplemental experiment on hedonic contrast in which participants judged the pleasantness of RT water after drinking a contrasting beverage (S1 Fig). The pleasantness of RT water is not affected by the sensory properties of the beverage they drank before hand.

#### Thirst quenching effect of a beverage with chemically-induced coldness

**Fig 2:**
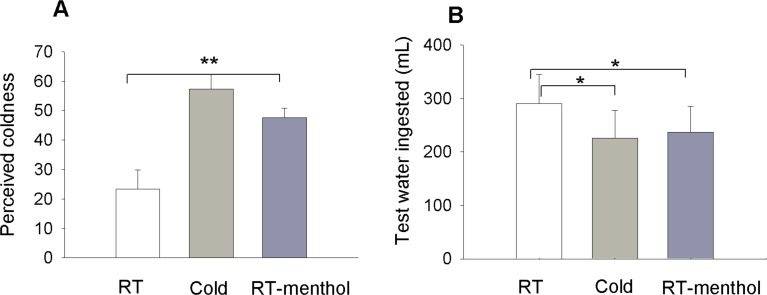
Effects of oral cold stimulation induced by menthol on thirst quenching. Figures display the volume of extra room temperature (RT) water ingested 15 min after consuming a 400 mL experimental beverage. In this study all the experimental beverages were presented with a pretreatment, either blank or containing 0.04% l-menthol. The pretreatment solutions were presented at RT. Legends correspond to the experimental beverage tested with mention of menthol when present in the pretreatment. All the experimental beverages were tested in duplicate. (A) Coldness ratings of the experimental beverages: RT water (21°C) vs Cold water (6°C) vs RT water (21°C) with menthol-pretreatment (n = 12 participants). (B) Volume of extra RT water consumed. Data are represented as mean +/- SEM. ** indicates statistical significance at p< 0.01 and * at p<0.05.

We examined the effect of oropharyngeal cold sensation on thirst reduction when chemically induced by l-menthol. The same protocol as above was used but here the 400 mL experimental beverages were preceded by a pre-treatment of l-menthol (see methods for details). We asked participants to rate the perceived coldness of the experimental beverage to validate the effects of the menthol pre-treatment [16]. There are, however, great individual differences in sensitivity to menthol’s cooling effects [17]. Since our experimental objective was to test the effects of illusory cold perception on thirst quenching, we restricted our analysis to participants for whom menthol caused RT water to be perceived as cool (cool ratings higher than 30 (moderately strong) on a LMS). The participants who were sensitive to menthol-cooling (Fig 2A) demonstrated a significant effect of l-menthol-pretreatment on thirst quenching that was comparable to the effect of physically cold water on thirst, despite the fact that they were actually drinking RT water as their experimental beverage (Fig 2B; p = 0.04 and 0.03 respectively). It is important to note that the test solution of RT water was not perceived as cool, since the effects of l-menthol had worn off by the time of testing (15 min time interval between drinking the experimental beverage and the presentation of the test jug). Thus, the thirst quenching properties of beverages were enhanced by perceived oral coldness whether elicited thermally or chemically.

### Study on estimated volume of beverages ingested when blinded to volume

**Fig 3:**
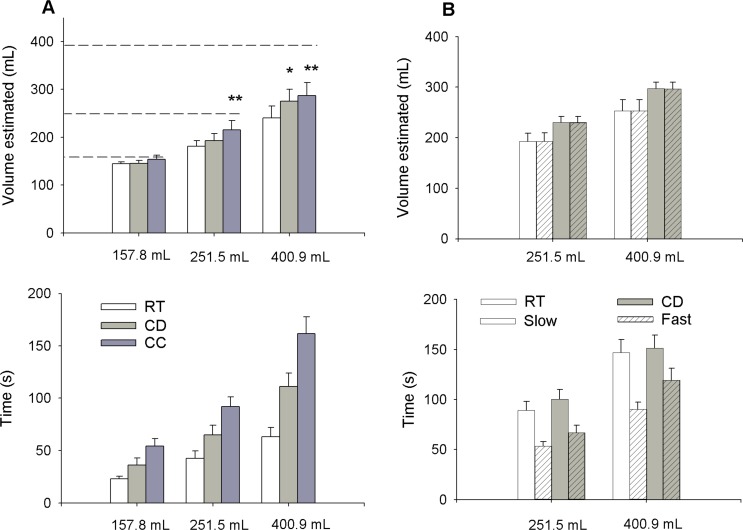
Estimated volume of beverages ingested when blind to the volume. Participants were asked to estimate how much they just ingested after drinking an experimental beverage presented in a lidded and opaque cup, through a straw without touching the cup. Legends and dashed lines correspond to actual volumes of the samples. Each condition was tested in triplicate. (A), Three beverages were tested: room temperature water (RT), cold water (CD) and cold carbonated water (CC). Upper panel displays ingested volumes estimated by participants and lower panel shows the time of completion to drink the whole sample. Data are represented as mean +/- SEM * indicates statistical significance at p<0.05 vs RT. (B), Participants did the same task with RT and cold water but at a forced rate (slow (10 bpm) or fast (20 bpm)). Upper panel displays ingested volumes estimated by participants and lower panel shows the time recorded to finish drinking the whole sample. Data are represented as mean +/- SEM. (n = 10 participants).

Cooling sensation and the bite of carbonation might help indicate the ingestion of water. To test this hypothesis, we asked participants to drink and then estimate the ingested volume of different beverages while they were unable to see or feel the beverages weight with their hand. Three beverages were tested: RT water, cold water, and cold-carbonated water, at three different volumes (157.8, 251.5 and 400.9 mL). For each separate test, the time of completion was recorded. Overall participants underestimated their fluid consumption, especially for the greater volumes ingested. Yet, they believed that they had consumed more fluid when the beverage was cold, and even more so when it was cold and carbonated by about 22% more, compared to the estimated volume ingested when the same sized beverage was at RT (Fig 3A, upper panel). Post hoc analysis volume estimated x experimental beverage using Fisher LSD method were performed for each level of volume offered ((157.8, 251.5 and 400.9 mL). At 400.9 mL, main effect of cold water compared to RT water was p = 0.29 and main effect of cold-carbonated water was p = 0.006.

The time required to finish a beverage tended to track the beverage’s perceived volume; participants finished the RT samples faster than the cold samples (Fig 3A, lower panel). It is, therefore, possible that total time spent drinking, rather than the sensations of cold or carbonation, influenced the perception and judgment of how much fluid had been ingested. We examined this question by repeating the study with both RT and cold water, but we asked the participants to drink the beverages at specified rates. The drinking rate was established by asking participants to sip once and swallow at each beat of a metronome. The “fast” rate was set at 20 beats per minute (bpm), which dictates one sip every three seconds, and the “slow” rate was set to half that speed, 10 bpm, or one sip every six seconds. Fig 3B shows that the rate of drinking the beverage had no impact on the estimation of how much water was ingested either at RT or when cold. [These data also confirm that volume estimations were greater when drinking cold water compared to RT water even when sipping at fixed rates (top panel)].

## Discussion

To investigate the role of oropharyngeal sensory signals on thirst quenching, we first determined the respective efficacies of several sensory trait(s) commonly manipulated in beverages, including cold, on thirst reduction. The preponderance of data on thirst quenching from beverages in humans has been subjective, based upon ratings of refreshment or thirst level, rather than objectively measuring volumes consumed [18]. Thirst quenching judgments are estimations of the actual thirst-reducing ability of a drink [19]; they may be greatly influenced by other factors, such as beverage flavor, palatability, and demand characteristics of the task, such as preconceived ideas and social norms about which beverage attributes should be thirst quenching. We designed our experiment to indicate thirst reduction operationally as a decrease in the volume of plain RT water consumed following the ingestion of a fixed-volume, experimental beverage. Thus, in order to compare the thirst quenching capacities of different experimental beverages directly, all tests of thirst involved the offering of the same test beverage: an excess of unflavored, ‘still’ (uncarbonated), room-temperature water to participants. The volume ingested of additional water assesses their residual thirst and, by extension, the thirst-quenching properties of the experimental beverage. This method enables us to compare our results directly with the literature on thirst quenching in non-verbal animals involving similar operational experiments.

We first examined the effect of cold temperature. We showed that participants drank significantly less RT water from the jug after a previous cold water beverage than after a RT water beverage, demonstrating that cold water reduced thirst more effectively than did the RT water. These results confirm the effect of cold stimulation on water intake in dehydrated humans, and are in agreement with studies showing greater reward value of a cold drink compared to a room temperature drink in water-deprived humans [20,21] and non-human animals [22].

Thirsty humans often prefer beverages that are both cold and carbonated including: mineral waters, seltzer, sodas, and beers. There is, however, no clear explanation for why this behavior is so common. We, therefore, investigated the effect of carbonation on thirst quenching. We found that like cold, carbonation enhances the thirst quenching properties of the beverage. Moreover, carbonation further reduces thirst beyond the effects of cold water when comparing cold water with cold, carbonated water. It is possible that the effect of carbonation on thirst satiation is due, at least in part, to gastric filling. But dissolved CO_2_ also elicits oral pungency [23], a very slight sourness, and has been shown to enhance the perception of cool sensations [24]. These sensations, as well as the lower pH of the commercial carbonated water (pH = 4.0 vs 5.4 for the still water), could be involved in the enhanced thirst quenching properties of the beverage. To test whether the lower pH/slight sourness of carbonated water affected thirst, we compared the effect of a matched-acidity (5 mM citric acid, pH = 4.0), cold water beverage to a plain, cold water beverage. Mild acidification at this level did not by itself appear to reduce thirst beyond the thirst quenching effects of a cold beverage. Therefore, the enhanced effect of cold, carbonated water on thirst quenching is not solely due to its lower pH or mild sourness. This result does not preclude possible effects of acids on thirst reduction at higher concentrations (lower pH), but it suggests that gastric gas filling, perceived carbonation bite in the oral cavity, and possible enhanced cool perception elicited by CO_2_ are central to its effect on thirst reduction. Carbonation and cold sensations are both mediated in part by pain/thermal receptors (nociceptors), especially transient receptor potential (TRP) channels, such as TRPM8, TRPV1, TRPA1 and TRPC5 [25–27]. It is possible that those sensations have an additive effect on perceived coldness, which further enhances thirst quenching, as observed in our study. In support of this hypothesis, Green [24] demonstrated that CO_2_ enhanced the perception of cool in the oral cavity from low temperature stimuli, and that low temperature stimuli increased the perceived irritation of CO_2_.

To further examine the contribution of cold-sensitive oropharyngeal receptors in thirst, we investigated the effect of chemically-induced oral cold sensations on thirst reduction. We used l-menthol’s ability to stimulate and sensitize the sensory cold receptor TRPM8 to test the hypothesis that a beverage illusorily perceived as cool, but physically at RT, quenches thirst more effectively than a RT beverage that is perceived to be at RT. We found that people sensitive to the cooling effect of menthol drank less water from the jug when the menthol pretreatment was applied. Thus, thirst reduction can be enhanced by perceived oral coldness whether elicited thermally or chemically.

We hypothesized that sensations from cold and carbonated water improve the thirst quenching properties of a beverage by increasing the sensory signals normally associated with drinking water. By analogy, a parallel process has been demonstrated for breathing, in which people can hold their breath significantly longer if they inhale cool air versus warm air [28], presumably because nasal and pharyngeal evaporative cooling is a correlated sensory cue to the volume of inspired air [29]. Thus, cooling sensations may help indicate both the volume of inspired air, as well as the volume of water ingested. To test this hypothesis, we asked participants to estimate the ingested volume of different beverages when blind to the actual volume. We found that judgments of the volume ingested were increased when the beverage was cold. Perceived volume was further increased when the beverage was cold and carbonated (> 20% increase). Here again it is possible that gastric filling by CO_2_ impacts the higher estimation of volume ingested. We believe that cold sensations and perhaps the bite of carbonation, which have sensory similarities to very cold water, are integrated sensory cues that inform the inferred volume of ingested water.

Water at temperatures well below oral temperature (~0 to 33°C) is perceived as cool or cold. And the lower the water temperature, the more “biting” a cold beverage will appear. CO_2_ is perceived as biting (or irritating) because it passes directly through cell membranes and acidifies tissues by forming carbonic acid. The tissue irritation from CO_2_ is not injurious; it mimics the pH changes associated with tissue damage, but does not actually harm tissue [30]. CO_2_ in beverages also enhances the perception of cool from low temperature liquids [24]. Overall, we believe CO_2_ increases the perception of drinking by enhancing the cool and biting signals of cold water. This, we conclude, is why cold-carbonated beverages are more thirst quenching than room temperature-still water. Related to this, Michou *et al*. recently demonstrated that cold temperatures and carbonation modulate water swallowing in humans [31]. Moreover, we demonstrated that the pre-exposure to l-menthol which chemically sensitizes TRPM8 cold receptors, among other TRP channels, can quench thirst, even when the beverage was at RT and the sensation of cool water was illusory. Thus, the perception of drinking water when thirsty appears to be mediated in part by cold, mildly irritating oral sensations, which, in turn, directly influence water intake. At the same time, these sensory manipulations not only influence thirst, but also result in the conscious perception that ingested water volume is greater when the water is either cold or cold-carbonated, irrespective of the rate at which it is ingested.

Our results are consistent with previous studies of the influence of cold sensations on drinking and licking in animals, which show that thirsty animals will strive not only to lick cold water over room temperature water, but even to lick streams of cold air or even solid pieces of cold metal that provide no fluids [1]. Our studies expand this idea both to include carbonation sensation that influences cold perception and to rule out strong influences from sour taste or acidity, sweetness, and astringency. Moreover, we show that cold and carbonation sensations alter the volume perception of participants, so that they believe they have ingested more water, than if they had ingested the same volume of room temperature, un-carbonated water, reinforcing the idea that thirst is quenched, in the short term, by the perception of drinking water, not necessarily by how much is consumed. These observations could explain the ubiquity of cold, carbonated beverages throughout the world, and are consistent with the idea that these beverages quench thirst more efficiently and are, therefore, more rewarding to thirsty people. Our data also have implications for groups who are known for clinical underhydration, such as laborers, soldiers, and the elderly, possibly due to their ignoring or cognitively mishandling important sensory cues that guide thirst and its quenching.

## Supporting Information

S1 Fig Participants (n = 20) rated the pleasantness of a cup (10 ml) of RT water 20 seconds after drinking a cup (10 ml) of either cold, cold carbonated or RT water, on a Labeled Hedonic Scale (Lim et al, Chem Senses 34: 739–751, 2009). Each condition was tested 5 times per each participant. Data are represented as mean +/- SEM.
(PDF)

S1 File Copy of Thirst Study Raw Data. This is the original raw data file.
(XLSX)

## References

[1] Rolls BJ, Rolls ET (1982) Thirst. Cambridge; New York: Cambridge University Press xiii, 194 p. p.

[2] Fitzsimons JT (1998) Angiotensin, thirst, and sodium appetite. Physiol Rev 78: 583–686. 9674690 10.1152/physrev.1998.78.3.583

[3] Arai SR, Butzlaff A, Stotts NA, Puntillo KA (2013) Quench the Thirst: Lessons From Clinical Thirst Trials. Biol Res Nurs. 10.1177/1099800413505900 PMC3989478 24136996

[4] Baylis PH (1987) Osmoregulation and control of vasopressin secretion in healthy humans. Am J Physiol 253: R671–678. 3318505 10.1152/ajpregu.1987.253.5.R671

[5] Seckl JR, Williams TD, Lightman SL (1986) Oral hypertonic saline causes transient fall of vasopressin in humans. Am J Physiol 251: R214–217. 3740301 10.1152/ajpregu.1986.251.2.R214

[6] Stricker EM, Hoffmann ML (2007) Presystemic signals in the control of thirst, salt appetite, and vasopressin secretion. Physiol Behav 91: 404–412. 17482653 10.1016/j.physbeh.2007.04.007

[7] Geelen G, Keil LC, Kravik SE, Wade CE, Thrasher TN, et al (1984) Inhibition of plasma vasopressin after drinking in dehydrated humans. Am J Physiol 247: R968–971. 6507654 10.1152/ajpregu.1984.247.6.R968

[8] Thompson CJ, Burd JM, Baylis PH (1987) Acute suppression of plasma vasopressin and thirst after drinking in hypernatremic humans. Am J Physiol 252: R1138–1142. 3591984 10.1152/ajpregu.1987.252.6.R1138

[9] Thrasher TN, Nistal-Herrera JF, Keil LC, Ramsay DJ (1981) Satiety and inhibition of vasopressin secretion after drinking in dehydrated dogs. Am J Physiol 240: E394–401. 7013497 10.1152/ajpendo.1981.240.4.E394

[10] Blair-West JR, Gibson AP, Woods RL, Brook AH (1985) Acute reduction of plasma vasopressin levels by rehydration in sheep. Am J Physiol 248: R68–71. 3918458 10.1152/ajpregu.1985.248.1.R68

[11] Bellows RT (1939) Time factors in water drinking in dogs. Am J Physiol 125: 87–97.

[12] Figaro MK, Mack GW (1997) Regulation of fluid intake in dehydrated humans: role of oropharyngeal stimulation. Am J Physiol 272: R1740–1746. 9227585 10.1152/ajpregu.1997.272.6.R1740

[13] Williams TD, Seckl JR, Lightman SL (1989) Dependent effect of drinking volume on vasopressin but not atrial peptide in humans. Am J Physiol 257: R762–764. 2529782 10.1152/ajpregu.1989.257.4.R762

[14] Salata RA, Verbalis JG, Robinson AG (1987) Cold water stimulation of oropharyngeal receptors in man inhibits release of vasopressin. J Clin Endocrinol Metab 65: 561–567. 3624414 10.1210/jcem-65-3-561

[15] Eccles R (2000) Role of cold receptors and menthol in thirst, the drive to breathe and arousal. Appetite 34: 29–35. 10744889 10.1006/appe.1999.0291

[16] Green BG (1985) Menthol modulates oral sensations of warmth and cold. Physiol Behav 35: 427–434. 4070414 10.1016/0031-9384(85)90319-1

[17] Cliff MA, Green BG (1994) Sensory irritation and coolness produced by menthol: evidence for selective desensitization of irritation. Physiol Behav 56: 1021–1029. 7824566 10.1016/0031-9384(94)90338-7

[18] McEwan J, JS C (1996) The sensory assessment of the thirst-quenching characteristics of drinks. Food Quality and Preference 7: 101–111.

[19] Brunstrom JM, Macrae AW (1997) Effects of temperature and volume on measures of mouth dryness, thirst and stomach fullness in males and females. Appetite 29: 31–42. 9268423 10.1006/appe.1997.0089

[20] Boulze D, Montastruc P, Cabanac M (1983) Water intake, pleasure and water temperature in humans. Physiol Behav 30: 97–102. 6836049 10.1016/0031-9384(83)90044-6

[21] Sandick BL, Engell DB, Maller O (1984) Perception of drinking water temperature and effects for humans after exercise. Physiol Behav 32: 851–855. 6494291 10.1016/0031-9384(84)90205-1

[22] Ramsauer S, Mendelson J, Freed WJ (1974) Effects of water temperature on the reward value and satiating capacity of water in water-deprived rats. Behav Biol 11: 381–393. 4413180 10.1016/s0091-6773(74)90667-1

[23] Wise PM, Wolf M, Thom SR, Bryant B (2013) The influence of bubbles on the perception carbonation bite. PLoS One 8: e71488 10.1371/journal.pone.0071488 23990956 PMC3749224

[24] Green BR (1992) The effects of temperature and concentration on the perceived intensity and quality of carbonation. Chemical Senses 17: 435–450.

[25] Vay L, Gu C, McNaughton PA (2012) The thermo-TRP ion channel family: properties and therapeutic implications. Br J Pharmacol 165: 787–801. 10.1111/j.1476-5381.2011.01601.x 21797839 PMC3312478

[26] Zimmermann K, Lennerz JK, Hein A, Link AS, Kaczmarek JS, et al (2011) Transient receptor potential cation channel, subfamily C, member 5 (TRPC5) is a cold-transducer in the peripheral nervous system. Proc Natl Acad Sci U S A 108: 18114–18119. 10.1073/pnas.1115387108 22025699 PMC3207667

[27] Wang YY, Chang RB, Liman ER (2010) TRPA1 is a component of the nociceptive response to CO2. J Neurosci 30: 12958–12963. 10.1523/JNEUROSCI.2715-10.2010 20881114 PMC2993877

[28] McBride B, Whitelaw WA (1981) A physiological stimulus to upper airway receptors in humans. J Appl Physiol 51: 1189–1197. 7298459 10.1152/jappl.1981.51.5.1189

[29] Burgess KR, Whitelaw WA (1988) Effects of nasal cold receptors on pattern of breathing. J Appl Physiol 64: 371–376. 3128527 10.1152/jappl.1988.64.1.371

[30] Kleinman RE (2008) Protection of the gastrointestinal tract epithelium against damage from low pH beverages. J Food Sci 73: R99–105. 10.1111/j.1750-3841.2008.00863.x 18803726

[31] Michou E, Mastan A, Ahmed S, Mistry S, Hamdy S (2012) Examining the role of carbonation and temperature on water swallowing performance: a swallowing reaction-time study. Chem Senses 37: 799–807. 10.1093/chemse/bjs061 22843761

