# Supplementary figures and images for: Oral Cooling and Carbonation Increase the Perception of Drinking and Thirst Quenching in Thirsty Adults
Source: PLoS One. 2016 Sep 29;11(9):e0162261. doi: 10.1371/journal.pone.0162261 (PMC5042416; doi:10.1371/journal.pone.0162261)

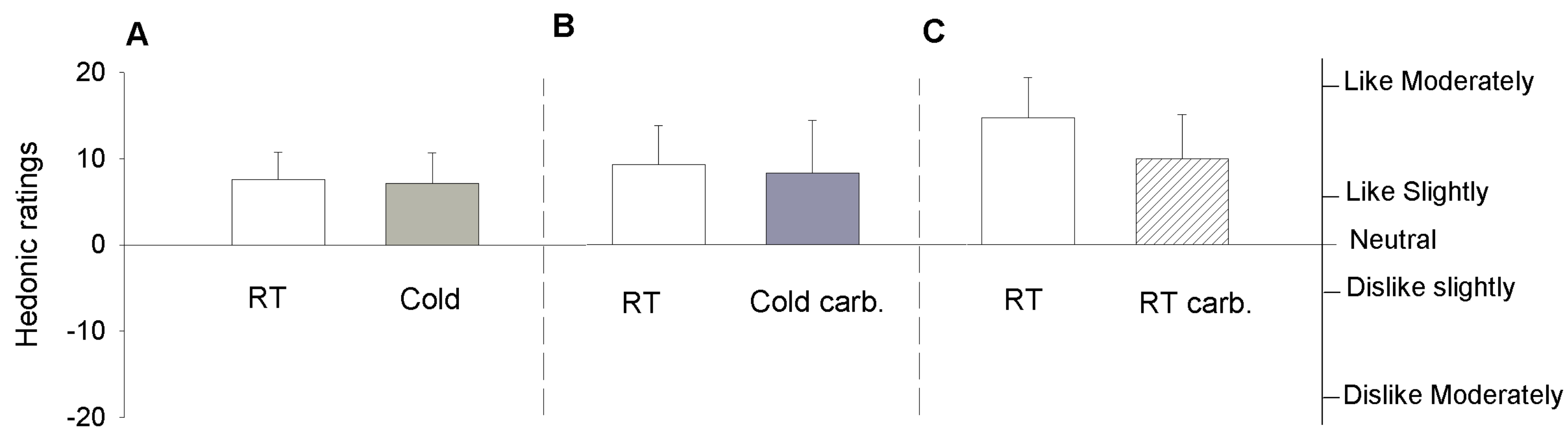

Supplement: S1 Fig — Each condition was tested 5 times per each participant. Data are represented as mean +/- SEM. (PDF) [file pone.0162261.s001.pdf]
